# Discovery of Small Molecules from *Echinacea angustifolia* Targeting RNA-Dependent RNA Polymerase of Japanese Encephalitis Virus

**DOI:** 10.3390/life12070952

**Published:** 2022-06-24

**Authors:** Pardeep Yadav, Sherif A. El-Kafrawy, Mai M. El-Day, Wejdan T. Alghafari, Arwa A. Faizo, Saurabh Kumar Jha, Vivek Dhar Dwivedi, Esam I. Azhar

**Affiliations:** 1 Department of Biotechnology, School of Engineering & Technology, Sharda University, Greater Noida 201310, India; par.yadav2011@gmail.com (P.Y.); saurabh.jha@sharda.ac.in (S.K.J.); 2 Center for Bioinformatics, Computational and Systems Biology, Pathfinder Research and Training Foundation, Greater Noida 201308, India; 3 Special Infectious Agents Unit-BSL-3, King Fahd Medical Research Center, King Abdulaziz University, Jeddah 21362, Saudi Arabia; saelkfrawy@kau.edu.sa (S.A.E.-K.); meldaly@kau.edu.sa (M.M.E.-D.); walghefari@kau.edu.sa (W.T.A.); aafaizo@kau.edu.sa (A.A.F.); 4 Department of Medical Laboratory Sciences, Faculty of Applied Medical Sciences, King Abdulaziz University, Jeddah 21362, Saudi Arabia; 5 Clinical Nutrition Department, Faculty of Applied Medical Sciences, King Abdulaziz University, Jeddah 21362, Saudi Arabia; 6 Department of Biotechnology Engineering and Food Technology, Chandigarh University, Mohali 140413, India; 7 Department of Biotechnology, School of Applied & Life Sciences (SALS), Uttaranchal University, Dehradun 248007, India; 8 Institute of Advanced Materials, IAAM, 59053 Ulrika, Sweden

**Keywords:** Japanese encephalitis virus, *Echinacea angustifolia*, Echinacoside, Echinacin, Rutin, Cynaroside, Quercetagetin 7-glucoside, Kaempferol-3-glucoside, docking, MD simulation

## Abstract

The Japanese encephalitis virus (JEV), a mosquito-borne flavivirus that causes viral encephalitis leading to neural damage, is a major threat in most Asian countries. The RNA-dependent RNA polymerase (RdRp) present in the viral genome is the key component for genome replication, making it an attractive target for antiviral drug development. In this study, the natural products from *Echinacea angustifolia* were retrieved for structure-based virtual screening against JEV–RdRp. The top six compounds (Echinacoside, Echinacin, Rutin, Cynaroside, Quercetagetin 7-glucoside, and Kaempferol-3-glucoside) were obtained based on the highest negative docking score, ADMET (absorption, distribution, metabolism, excretion, and toxicity), and molecular interaction. The computational analysis of these selected compounds against the co-crystallized ligands, i.e., ATP and GTP, were performed. Further, 100 ns molecular dynamic simulation and post-free binding energy calculation of all the selected compounds complexed with JEV–RdRP were performed to check the stability of the complexes. The obtained results showed considerable stability and intermolecular interaction with native ligand-binding site residues of JEV–RdRp. Hence, selected natural compounds are admissible inhibitors of JEV–RdRp protein and can be considered for future antiviral drug development studies.

## 1. Introduction

Japanese encephalitis (JE) is the most commonly diagnosed epidemic encephalitis—a vector-borne acute central nervous system infection, caused by the Japanese encephalitis virus (JEV) that belongs to the genus Flavivirus of the family Flaviviridae and comprises five genotypes (GI–GV) [1,2,3]. JEV is transmitted to humans by the bite of infected mosquitoes (*Culex* spp.) and causes severe neurological manifestations [4,5]. JEV is an endemic in eastern and southern Asia countries and Australia, with fatality rates of 20–30% and life-long neurological impairments and sequelae among one-half of the survivors [6,7,8,9,10]. Nearly 68,000 clinical cases of JEV infection and approximately 20,000 deaths are reported annually in many Asian countries, and its transmission is endemic in 24 countries of Southeast Asia and Western Pacific regions [11,12,13]. According to World Health Organization (WHO), more than 3 billion people are at risk of JEV infection globally [11,12,13]. 

JEV is characterized as a positive-sense single-stranded RNA (+ssRNA) genome encapsulated by an icosahedral-shaped viral capsid. The RNA genome of JEV (≈11 kb) is modified at the 5′ end cap with an m^7^GpppAm structure and mimics cellular mRNAs, except for lacking the polyadenylated tail [14]. The viral genome encodes a single 370 kDa polyprotein, which is subsequently cleaved by a combination of viral and host proteases to yield three structural (capsid, membrane, and envelope) and seven nonstructural (NS: NS1, NS2A, NS2B, NS3, NS4A, NS4B, and NS5) proteins [14,15]. Among the seven NS proteins, NS5 is the largest (~105 kDa) and conserved viral protein—housing a C-terminal RNA-dependent RNA polymerase (RdRp) and an N-terminal guanylyltransferase (GTase) and methyltransferase (MTase) activity—and is associated with other viral proteins, host factors, and viral RNA to form the replication complex (RCs) [16,17]. The JEV NS5–RdRp domain (JEV–RdRp) replicates the genomic RNA into the uncapped minus-strand RNA, which is subsequently used as a template to produce a large excess of the positive-sense viral RNA genome [18]. The JEV NS5 MTase (JEV^MTase^) domain then performs both guanine N-7 and ribose 2′-OH methylations for the capping of the newly synthesized positive-sense RNAs [19,20]. Moreover, the JEV–RdRp domain is involved in the synthesis of both negative- and positive-strand RNAs during viral genome replication and translation [21]. As the JEV–RdRp domain is required for RNA synthesis in viral replication and lacks a similar protein in host cells, it has been demonstrated as a promising target for antiviral drug development against the JEV infection [14,22].

To tackle the JEV infection, both inactivated and live-attenuated vaccines have been progressively designed and utilized to prevent the JEV infection [23,24]. Unfortunately, regardless of a licensed vaccine to prevent JEV infection, infections strike annually due to a denial of complete coverage and access [25,26,27,28,29]. Several other efforts were also made in laboratories globally to identify an effective and safe therapeutic agent demonstrating an anti-JEV activity, including N-nonyl-deoxynojirimycin [30], dehydroepiandrosterone [31], N-methylisatin-beta-thiosemicarbazone derivative (SCH 16) [32], indirubin [33], manidipine [34], chlorpromazine [35], etanercept [36], minocycline [37], ouabain, and digoxin [38], but none of them is yet approved as antiviral against JEV infection treatment. The lack of efficient and approved antiviral agents in the prevention and treatment of the JEV infection, therefore, demands a potential antiviral to reduce JEV-infection-associated morbidity and mortality rates and continue to increase in global distribution.

In drug discovery pipelines, medicinal plants have become the popular source of natural compounds with high structural diversity against viral infections [39,40,41,42,43]. Several medicinal plants or natural compounds have shown promising effects against JEV [33,44,45,46,47]. *Echinacea angustifolia* (*E. angustifolia*) is one of the most well-known medicinal plants in its genus, which has been studied for medicinal properties, including antibacterial, anti-inflammatory, and antiviral activity properties [48,49,50,51,52,53]. However, to the best of our knowledge, the phytoconstituents of *E. angustifolia* are not yet demonstrated for the antiviral activity against JEV. Therefore, the present study was designed to explore the potential of natural compounds as therapeutic against the JEV infection via inhibition of the JEV–RdRp domain using molecular simulations, including structure-based virtual screening, molecular docking, and molecular dynamics (MD) simulations, as well as free energy calculations. 

## 2. Methodology

### 2.1. Receptor and Ligand Collection

The three-dimensional (3D) crystal structure of the JEV–RdRp complexed with guanosine-5′-triphosphate (GTP) (PDB ID: 4HDG) [22] as a receptor was downloaded from the PDB database (https://www.rcsb.org/) [54]. Additionally, a total of 53 natural compounds, reported in *Echinacea angustifolia*, were collected from the literature, and their 3D atomic coordinates were retrieved from the PubChem database (https://pubchem.ncbi.nlm.nih.gov/) [55] to form the ligand library for further computational analysis. 

### 2.2. Virtual Screening, Redocking Analysis, and ADME

For the structure-based virtual screening, the JEV–RdRp domain was processed and minimized using the Dock Prep tool in UCSF Chimera [56], with default parameters. Here, the receptor molecule was prepared by removing the native ligand, water molecules, heteroatoms, and non-polar hydrogen atoms, along with the addition of Gasteiger charges and polar hydrogen atoms, followed by structure minimization, using 100 and 50 steps of steepest descent and conjugate gradient methods, respectively. Furthermore, for the ligand library preparation, i.e., the collected natural compounds of *Echinacea angustifolia* and structure-based virtual screening, PyRx0.8 (Virtual Screening Tool) [57] was used, under default parameters. Herein, each ligand was initially added for Gasteiger partial charge, and energy was minimized under MMFF94 force field using default steepest descent and conjugate gradient with 1000 steps, followed by the addition of polar hydrogens, as reported earlier [58]. The prepared ligands were docked in the binding pocket of JEV–RdRp covering essential residues (Lys^463^, Lys^471^, Arg^474^, Asp^541^, Ser^604^, Asp^668^, Arg^734^, Arg^742^, Ser^799^, Try^800^, and Ser^801^) in the docking grid 39.3513 Å × 29.2100 Å × 28.2521 Å and center at −37.5873 Å, −5.1747 Å, −35.1844 Å along the X, Y, and Z axes. 

Subsequently, the top 10 ranked ligands based on binding scores were selected for redocking analysis by comparison to the ATP and GTP as reference molecules using the Chimera-AutoDock Vina Plugin setup [56,59]. Herein, the processed protein structure was docked with the selected compounds in the same docking pocket as considered for virtual screening (center coordinates: −37.5873 Å, −5.1747 Å, −35.1844 Å; grid size: 39.3513 Å × 29.2100 Å × 28.2521 Å) under default parameters. Then, at least 10 docked poses were generated for each ligand, and the docked conformations with the highest negative docking scores and least root-mean-square deviation (RMSD) values (by default 0 in AutoDock Vina) were extracted for binding pose and intermolecular interaction analysis, under default parameters, in Maestro tool of Schrödinger suite 2018-4 [60]. All 3D and 2D figures were generated in Maestro tool of Schrödinger suite2018-4 [60]. 

SwissADME online server (http://www.swissadme.ch) was used to understand the properties related to the pharmacokinetics and drug likeliness of the selected compounds such as absorption, distribution, metabolism, and excretion (ADME) [61]. The bioavailability is another important parameter that makes any drug a promising therapeutic, and therefore, several parameters were observed during the ADME analysis such as blood–brain barrier (BBB), permeability, and cytochrome inhibition activities.

### 2.3. Molecular Dynamics Simulation 

The molecular dynamics (MD) simulations for the selected best poses of the top six protein–ligand complexes were performed using with academic Maestro-Desmond v5.6 suite [62,63]. Initially, the docked complex was placed in the center of the orthorhombic box (10 Å  ×  10 Å  ×  10 Å) and amended with an explicit TIP4Pwater model using the system builder module. Thereafter, the complete system was neutralized using the counter sodium and chloride ions, while being aced at a distance of 20 Å around the docked ligand in the binding pocket of protein, and then minimized under default parameters using the minimization tool. Next, the whole system was simulated using an NPT ensemble, maintained by the Nose–Hoover thermostat and the Martyna–Tobias–Klein barostat method [64], with the temperature set at 300 K and pressure set at 1.013 bar under default parameters. For the simulations, the cutoff radius in Coulomb interactions was set at 9.0 Å, and the long-range electrostatic interactions were computed via the particle mesh Ewald method [65]. Each complex was simulated for 100 ns under optimized potentials for liquid simulations (OPLS)-2005 force-field parameters, and a total of 5000 frames were saved for analysis. The generated JEV–RdRp trajectories with selected ligands were analyzed for statistical parameters, including root-mean-square deviation (RMSD) and root-mean-square fluctuation (RMSF) analysis, and intermolecular interactions (protein–ligand contact mapping) as a function of 100 ns using the Simulation Interaction Diagram (SID) tool implemented in free academic Desmond module with Maestro-Schrödinger suite 2018–4 interface [62,63].

### 2.4. Post-Simulation Analysis

#### 2.4.1. Essential Dynamics

The essential dynamics, defined in terms of principal component analysis (PCA), to study the dynamic motion of the protein was assessed in the presence of docked ligands from the respective MD simulation trajectories using the Bio3d package [66]. For this purpose, a total of 5000 frames of the Cα atoms from the respective MD simulation trajectories were superimposed on the initial pose to reduce the root-mean-square variations between similar residues by utilizing the fit.xyz function. These superimposed poses were then analyzed to generate respective plots of PCA components from each simulation trajectory using the pca.xyz function of the plot (pc) in the Bio3d package [66]. 

#### 2.4.2. Prime MM/GBSA Binding Free Energy Calculations 

To calculate the binding free energy and ligand strain energy for the docked poses, the endpoint MMGBSA calculation was applied to the complete 100 ns MD simulation trajectories using the thermal_mmgbsa.py python script in the Prime MMGBSA module of the Schrödinger suite (Schrödinger Release 2020-4: Prime, Schrödinger, LLC, New York, NY, USA, 2020), where each snapshot was treated for the removal of solvent and ions, as well as split into individual protein and ligand conformation for the free energy calculation. The net free binding energy (Δ*G*) was calculated using the following equation:

Δ*G*_Bind_ = Δ*G*_complex__(__minimized__)_ − (Δ*G*_receptor__(__minimized__)_ + Δ*G*_ligand__(__minimized__)_) (1)

where Δ*G*_Bind_ denotes the binding free energy; ∆*G*_complex_ indicates the binding free energy of the complex; ∆*G*_Receptor_ and ∆*G*_Ligand_ exhibit the energy for receptor and ligand, respectively. Finally, the computed binding free energy, along with energy dissociation components, was provided as average with the standard deviation for each simulation trajectory. 

## 3. Results and Discussion 

### 3.1. Structure-Based Virtual Screening and ADME

The natural products reported in the *E. angustifolia* were collected from the reported literature, and the respective 3D structures were collected retrieved from the PubChem database. The collected compounds were used as ligand libraries for the structure-based virtual screening against the nucleotide GTP-binding pocket in the crystal structure of JEV–RdRp (PDB ID: 4HDG). The top conformations of the screened ligands exhibited binding affinity ranging from −11.1 to −1.9 kcal/mol, targeting the binding pocket of the selected JEV–RdRp protein (Table S1). Thus, the top docked poses of the first six compounds—namely, Echinacoside, Echinacin, Rutin, Cynaroside, Quercetagetin 7-glucoside, and Kaempferol-3-glucoside—with significant binding scores (>−9 kcal/mol) were considered for the ADME/Tox and redocking analysis to find drug-likeness properties and the most ideal docking conformation, respectively, in the selected binding pocket of the viral protein. 

Drug likeness and pharmacological characters are considered to be important factors for understanding the medicinal application of the compounds. Therefore, the ADME analysis of the top six compounds, i.e., Echinacoside, Echinacin, Rutin, Cynaroside, Quercetagetin 7-glucoside, and Kaempferol-3-glucoside, were predicted using SwissADME, resulting in the properties related to the pharmacokinetics and toxicity (Table S2). All of the selected compounds were found to be non-inhibitors of Cytochrome P450 2D6 (CYP2D6), as the inhibition of CYP2D6 leads to drug–drug interactions. Additionally, each of these natural compounds was found to be impermeable to the Blood–brain barrier (BBB) (Table S2). Based on these findings, as well as other properties such as pharmacokinetics and drug-likeness, the selected compounds have considerable medicinal properties.

### 3.2. Redocking and Molecular Contact Analysis

**Figure 1:**
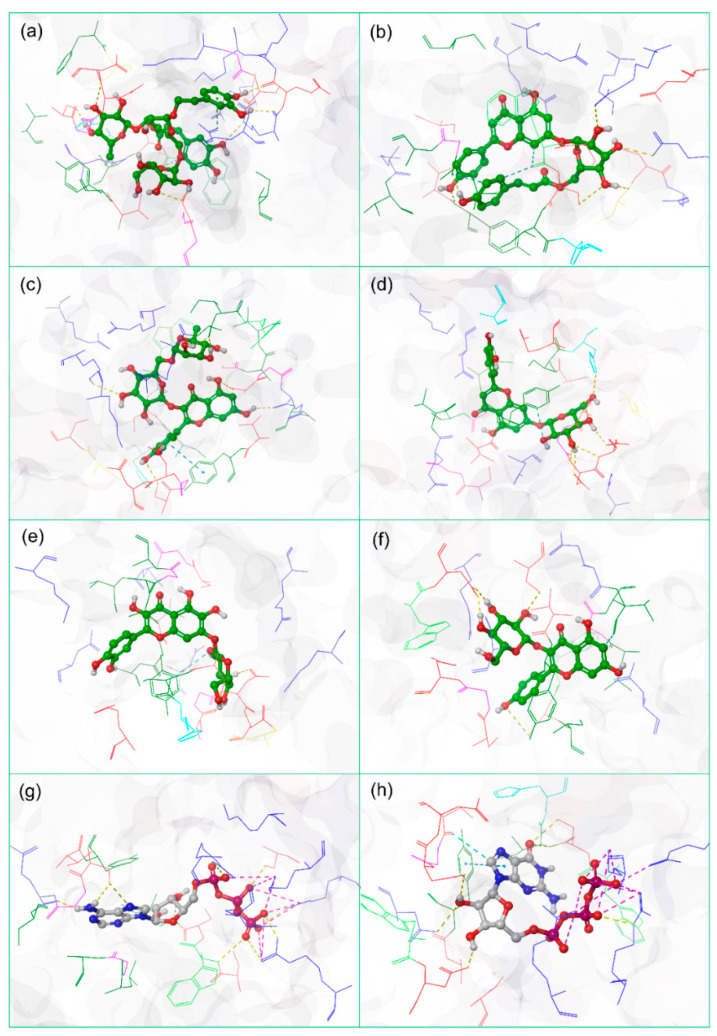
Molecular redocked poses of the selected natural compounds: (**a**) Echinacoside, (**b**) Echinacin, (**c**) Rutin, (**d**) Cynaroside, (**e**) Quercetagetin 7-glucoside, and (**f**) Kaempferol-3-glucoside, with comparison to the reference compounds (**g**) ATP and (**h**) GTP in the selected binding pocket of JEV–RdRp showing intermolecular interactions with residues extracted at 4 Å around the docked ligand.

From the virtual screening, six potential natural compounds—Echinacoside, Echinacin, Rutin, Cynaroside, Quercetagetin 7-glucoside, and Kaempferol-3-glucoside—were selected for redocking in the selected binding pocket of JEV–RdRp against ATP and GTP as reference compounds using AutoDock Vina. Afterward, the docked poses with the highest negative docking energy values corresponding to zero RMSD values for each natural compound were considered for further computational analysis (Figure 1). In this study, among the selected natural compounds, Echinacoside docked with JEV–RdRp showed maximum docking energy (−11.1 kcal/mol), while JEV–RdRp–Kaempferol-3-glucoside docked complex was noted for lowest docking scores (10 kcal/mol) by comparison to reference compounds ATP (8.6 kcal/mol) and GTP (−9.0 kcal/mol). Additionally, a substantial number of intermolecular interactions, including hydrogen bond formation, π–π stacking, π–cation, hydrophobic, polar, negative, positive, glycine, and salt bridge interactions were also noted in the docked complexes by comparison to the reference docked complexes (Table 1, Figure S1). Therefore, the calculated docking scores and intermolecular contact profiling between the docked natural compounds and JEV–RdRp indicates the stability of the respective docked complexes. 

### 3.3. Molecular Dynamics Simulation Analysis 

**Figure 2:**
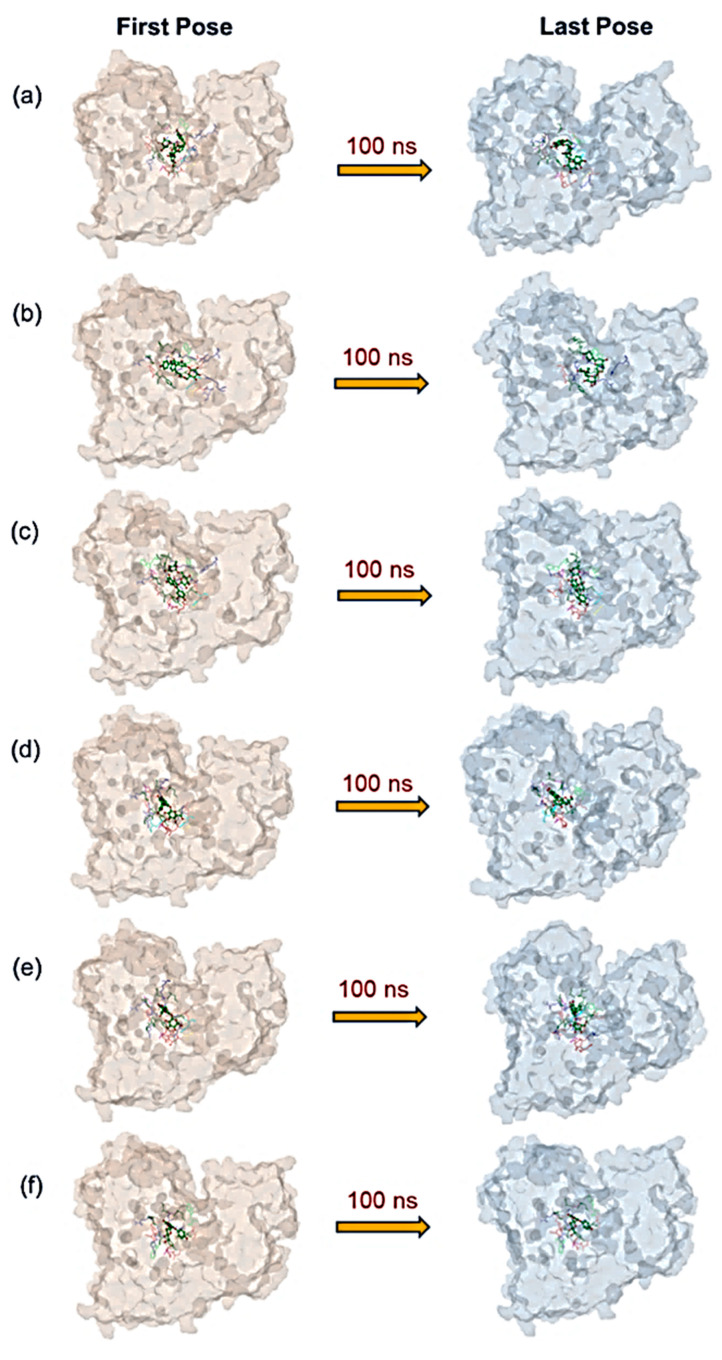
Representation of docked natural compounds: (**a**) Echinacoside, (**b**) Echinacin, (**c**) Rutin, (**d**) Cynaroside, (**e**) Quercetagetin 7-glucoside, and (**f**) Kaempferol-3-glucoside, in the selective binding pocket of JEV–RdRp before and after the 100 ns MD simulation.

A molecular dynamics simulation is used to predict the stability of protein–ligand complexes with respect to simulation intervals. In this study, the molecular dynamics simulation was analyzed in terms of the last pose from the simulation trajectory, protein and protein-fit-ligand root-mean-square deviation (RMSD), protein and protein-fit-ligand root-mean-square fluctuation (RMSF), and protein–ligand contact mapping as a function of 100 ns interval. Figure 2 shows the last poses of the MD trajectory were extracted and compared with the initially docked poses to monitor the relative occupation of the docked ligands in the protein structure. Notably, all of the docked natural compounds showed relatively substantial residence in the selective pocket of the JEV–RdRp as a function of 100 ns MD simulation interval, except for an acceptable deviation in the ligand conformations, which was noted against reference compounds (Figure S2). These results suggested the docked natural compounds as substantial inhibitors by comparison to the reference compounds, i.e., ATP and GTP.

**Figure 3:**
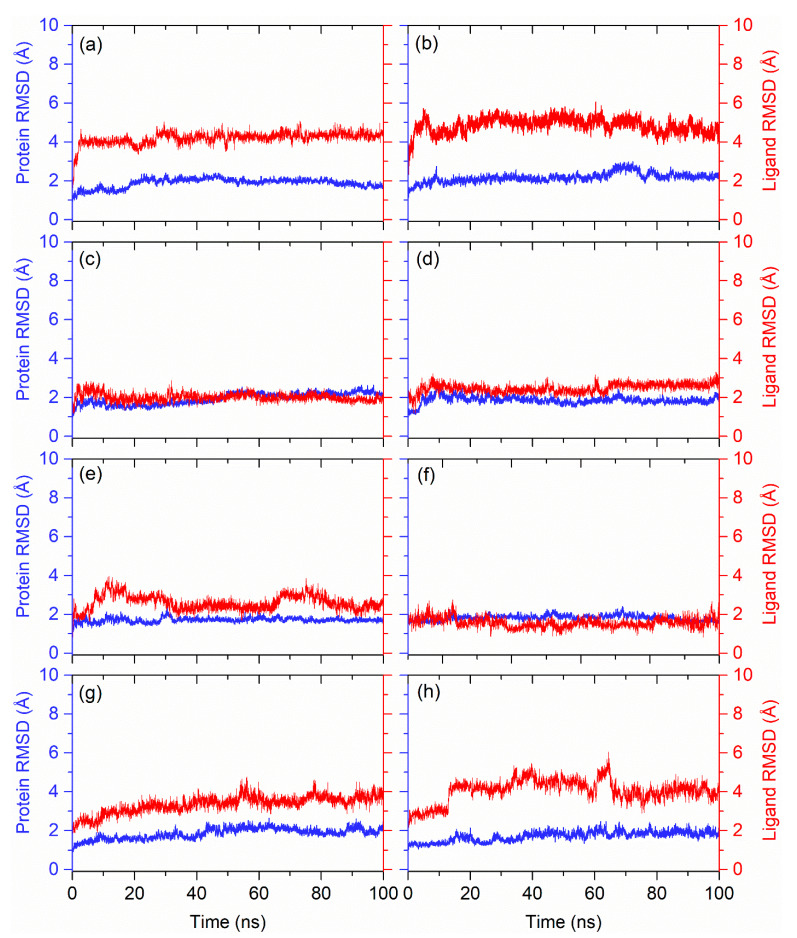
RMSD plots for the docked complexes of JEV–RdRP with selected natural compounds: (**a**) Echinacoside, (**b**) Echinacin, (**c**) Rutin, (**d**) Cynaroside, (**e**) Quercetagetin 7-glucoside, and (**f**) Kaempferol-3-glucoside, with comparison to the reference compounds (**g**) ATP and (**h**) GTP.

Furthermore, the calculated RMSD for the protein in all of the docked complexes with natural compounds indicated acceptable deviations (<2 Å) with respect to 100 ns MD simulation by comparison to reference compounds (Figure 3). These observations were also supported by the calculated RMSF values (Figure S3) for the protein structures docked with respective compounds, except for occasional residual higher fluctuations (<5 Å) that were also noted in the terminal regions and residues forming direct contact or adjacent residues to the interacting residues with the docked ligands. Moreover, the calculated protein-fit-ligand RMSD values showed considerable deviations (<4.5 Å) (Figure 3) throughout the 100 ns simulation interval against reference compounds, i.e., ATP (<4.0 Å) (Figure 3g) and GTP (<4.0 Å) (Figure 3h). Notably, among the selected natural compounds, Rutin, Cynaroside, and Kaempferol-3-glucoside showed the most substantial stability and equilibrium in protein-fit-ligand values as a function of 100 ns against reference compounds, i.e., ATP and GTP. Moreover, the calculated RMSF values also showed variation within 2 Å (Figure S4) during the 100 ns, supporting the observed stability of the docked ligands with the JEV–RdRp during MD simulation. 

Moreover, the protein–ligand contacts were also extracted from the 100 ns MD simulation trajectories for all of the docked complexes. Specifically, each type of intermolecular interaction, hydrogen bonding, hydrophobic interactions, water bridge formation, and ionic interaction was extracted and plotted as a fraction of the total molecular contact formed during the 100 ns interval for all the simulated complexes.

**Figure 4:**
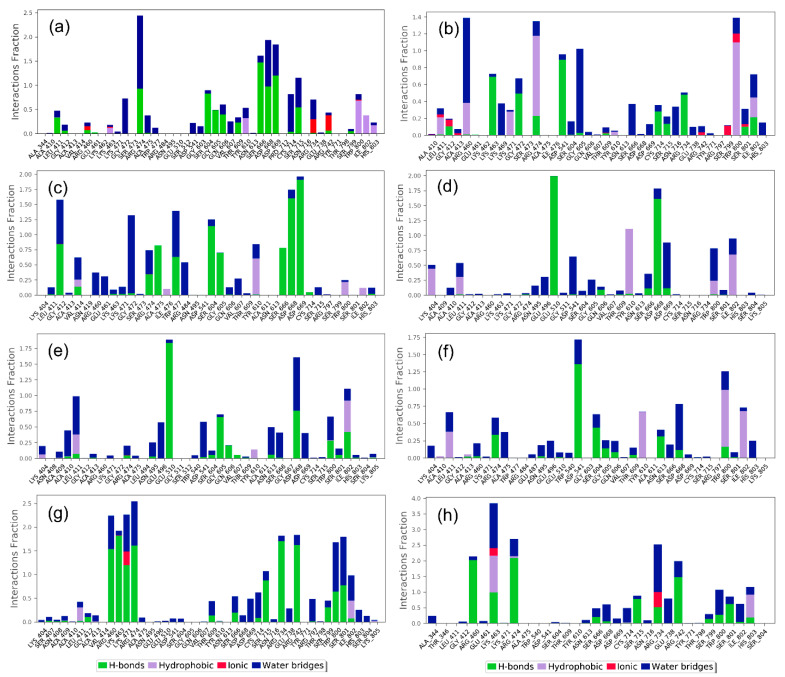
Protein–ligand interaction plots for the selected natural compounds: (**a**) Echinacoside, (**b**) Echinacin, (**c**) Rutin, (**d**) Cynaroside, (**e**) Quercetagetin 7-glucoside, and (**f**) Kaempferol-3-glucoside by comparison to the reference compounds (**g**) ATP and (**h**) GTP, docked with the JEV–RdRp; the plots were extracted from the 100 ns MD simulation interval.

In the case of the Echinacoside–JEV–RdRp docked complex, it exhibited hydrogen bond formation for more than 100% of simulation time in Ser^666^ and Asp^669^ residues, while Trp^800^ and Ile^802^ residues showed 50% and 35% of total simulation time for the hydrophobic interaction with the docked ligand; Gly^472^, Cys^714^, and Arg^474^ residues participated in water bridges formation, in which Arg^474^ residue showed more than 100% of water bridge formation during the simulation time in addition to a significant H-bond. Additionally, Arg^734^ and Arg^742^ residues formed an ionic bond for more than 20% simulation time (Figure 4a). Likewise, the Echinacin–JEV–RdRp complex exhibited hydrogen bond interaction in Asp^541^ and Lys^463^ residue for 90% and 70% of total simulation time; Trp^800^ and Arg^474^ residues were also noted to form hydrophobic interaction for 100% of simulation time. Gly^605^ and Arg^460^ residues showed more than 80% of the total interaction fraction in water bridge formation during the simulation period (Figure 4b). Additionally, protein–ligand contact analysis of Rutin–JEV–RdRp showed a high contribution of Asp^668,^ Asp^669^, and Ser^604^ in hydrogen bond formation (100%) during the total simulation interval. Tyr^610^ residue participated in hydrophobic interaction for more than 50% of the simulation time. A water bridge was formed by Gly^412^, Trp^477^, and Arg^484^ for more than 50% of simulation time and by Gly^412^ for 100% of simulation time (Figure 4c). Furthermore, the Cynaroside–JEV–RdRp complex exhibited a hydrogen bond with Glu^510^ and Asp^668^ residues for 100% of the simulation time. Tyr^610^ also showed the hydrophobic interaction with the docked ligand at a 100% of simulation interval. Asp^541^ and Asp^669^ residues were involved in water bridge formation for more than 50%, along with the hydrogen bond (Figure 4d). In comparison, in the Quercetagetin 7-glucoside–JEV–RdRp complex, Glu^510^ residue showed more than 100% of the total interaction fraction in hydrogen bond formation during the simulation interval, and Gly^605^ and Asp^668^ residues participated in hydrogen bond formation for more than 50% of simulation time; Asp^668^ also participated in water bridge formation (50%) during the simulation interval. Ile^802^ residue was noted for hydrophobic interaction with the docked ligand for more than 50% of the simulation time (Figure 4e). In the protein contact mapping analysis of the Kaempferol-3-glucoside–JEV–RdRp complex, Asp^541^ exhibited more than 100% of hydrogen bond formation during the 100 ns simulation time, whereas Tyr^610^, Trp^800^, and Ile^802^ residues showed 50% of hydrophobic interaction during the total simulation period. Notably, Asp^668^ residue showed more than 50% of the water bridge interaction along with the hydrogen bond (Figure 4f). In contrast, the protein–ligand mapping of the JEV–RdRp with its native ligand ATP exhibited more hydrogen bond formation for 100% of simulation time with Arg^460^, Lys^463^, Lys^471^, Arg^474^, and Arg^734^ residues, along with water bridge formation (50% of the interaction fraction). Ile^802^ and Lys^471^ residues participated in hydrophobic (50%) and ionic interaction (30%) during the 100 ns simulation period (Figure 4g). When the protein–ligand mapping of the JEV–RdRp with its native ligand GTP was analyzed, Arg^460^, Arg^474^, and Arg^742^ participated in hydrogen bond formation for more than 100%, in addition to the water bridge interaction of the total simulation time. Additionally, Lys^463^ and Arg^734^ exhibited hydrophobic and ionic bonds for more than 90% and more than 50% of the total interaction fraction (Figure 4h). The solvent accessible surface area (SASA) of the selected natural compound and the reference molecules were also studied to track the protein’s flexibility, stability, and folding in the presence and absence of ligands (Figure S5) [43]. 

Collectively, the analysis of the MD simulations supported the selected natural compounds as inhibitors of JEV–RdRp against the reference compounds, i.e., ATP and GTP. Hence, based on the statistical analysis and intermolecular interaction profiling of the docked natural compounds in the selected pocket of JEV–RdRp, the potential ligands can be placed in the order of Rutin, Cynaroside, Kaempferol-3-glucoside, Echinacoside, Quercetagetin 7-glucoside, and Echinacin, exhibiting the most stability with the JEV–RdRp protein. 

### 3.4. Post-Simulation Analysis 

#### 3.4.1. Principal Component Analysis

**Figure 5:**
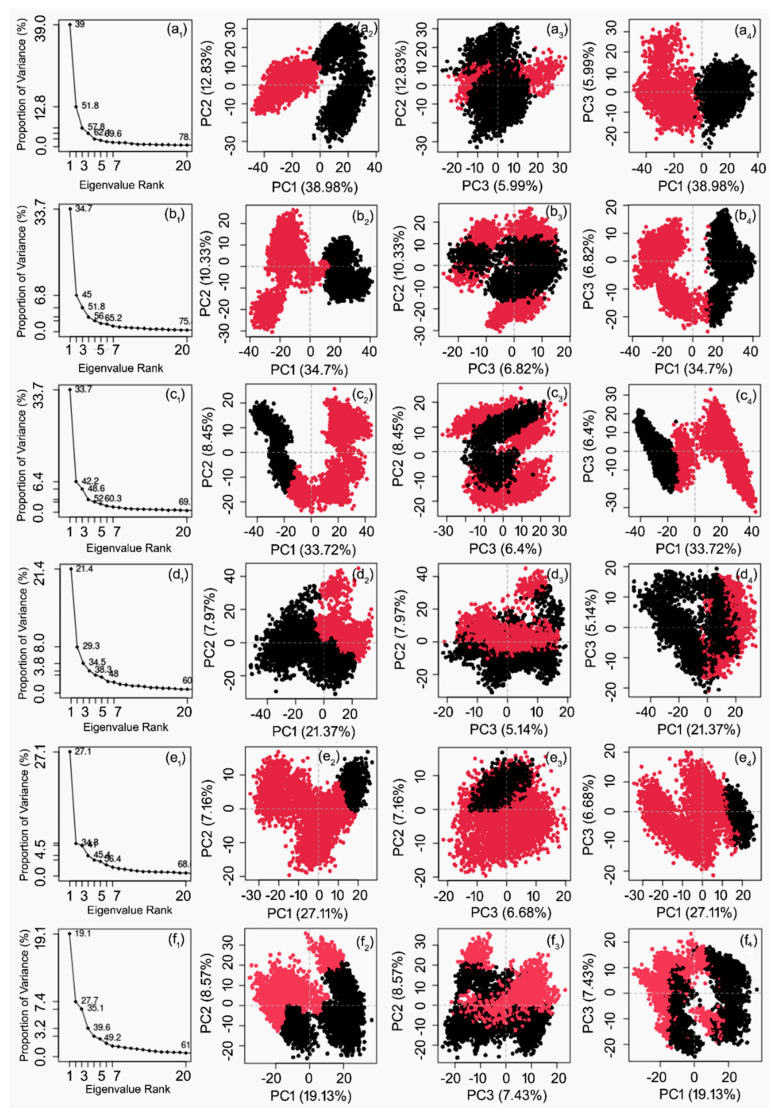
Principal component analysis for the generated molecular dynamics trajectories of JEV–RdRp docked with selected natural compounds: (**a**) Echinacoside, (**b**) Echinacin, (**c**) Rutin, (**d**) Cynaroside, (**e**) Quercetagetin 7-glucoside, and (**f**) Kaempferol-3-glucoside. The percentage of total mean square displacement of residual positional variations recorded in each dimension is categorized by equivalent eigenvalues or PCs.

Studying the essential dynamics based on principal component analysis provides a method to collect the domain dynamics and displacement of atoms in the protein, required for biological function. Figure 5 shows the percentage of variance (%) for the calculated mean square position variations in the covariance matrix as a function of extracted 20 eigenmodes from the respective MD simulation trajectories of the docked complexes. Notably, all of the JEV–RdRp structures docked with natural compounds relatively showed similar drops in eigen fraction values by comparison to the protein structures docked with reference compounds GTP but not ATP (Figure 5 and Figure S6). However, no significant changes in the eigen fraction were observed from 6 to 20 eigenvalues. These results indicated the significant conformational changes in the viral protein structure docked with natural compounds to attain the most stable complex formation against reference compounds.

Furthermore, the first three principal components were extracted and plotted to analyze the cluster motion in the protein structure as a function of 100 ns MD simulation. Notably, all of the complexes of JEV–RdRp docked with natural compounds showed higher cluster distributions of −40 and 40 along the direction of PC1, −30 and 30 along the direction of PC2, and −30 and 30 along PC3 by comparison to reference protein docked with ATP and GTP, which showed reduced and overlapped clusters along −30 and 20 and −40 and 20 coordinates for all three PCs (Figure 5 and Figure S6). These observations suggested that the docked natural compounds have the potential to induce conformational fluctuations in JEV–RdRp to distribute its biological function for the replication of the JE virus.

#### 3.4.2. Binding Free Energy Calculation

**Figure 6:**
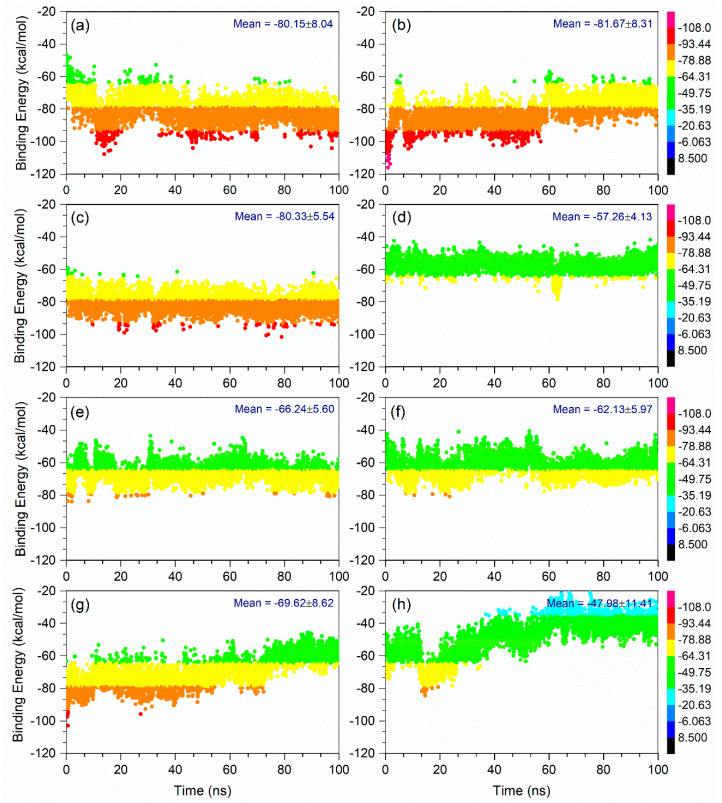
Binding free energy calculated on the 5000 poses generated during 100 ns MD simulation of the selected natural compounds: (**a**) Echinacoside, (**b**) Echinacin, (**c**) Rutin, (**d**) Cynaroside, (**e**) Quercetagetin 7-glucoside, and (**f**) Kaempferol-3-glucoside, (**g**) Adenosine Triphosphate, (**h**) Guanosine-5′-Triphosphate, docked with JEV–RdRp protein.

All of the generated MD simulation trajectories created as a function of 100 ns interval were treated for free binding energy calculation using MM/GBSA method against reference complexes. Figure 6 shows high and low binding energy values for all the docked complexes with respect to time. Notably, Echinacoside, Echinacin, and Rutin were found to exhibit the highest binding energy values (>−100 kcal/mol) for some poses, with net binding free energy values of −80 ± 8.04, 81.67 ± 8.31, and 80.33 ± 5.54 kcal/mol, respectively. In comparison, the other three natural compounds showed mean binding free energy between 66 to 57 kcal/mol. Interestingly, the calculated binding free energy values were relatively higher than those of the reference complexes, i.e., ATP (69.62 ± 8.62 kcal/mol) and GTP (47.98 ± 11.41 kcal/mol) (Figure 6). Additionally, net energy dissociation components—namely, Δ*G*_Bind Coulomb_, Δ*G*_Bind Covalent_, Δ*G*_Bind Hbond_, Δ*G*_Bind Lipo_, Δ*G*_Bind Packing_, Δ*G*_BindSolvGB_, and Δ*G*_Bind vdW_—were determined using the Prime MM/GBSA method and were also studied on the complete 100 ns MD trajectories of each complex. Interestingly, substantial contributions of Δ*G*_Bind Coulomb_, Δ*G*_Bind Lipo_, and Δ*G*_Bind vdW_ interactions were noted for favorable energy, while Δ*G*_Bind Covalent_ and Δ*G*_BindSolvGB_ exhibited unfavorable energy to the net binding free energy for all of the protein–ligand complexes during the 100 ns MD simulation (Table 2). Altogether, the natural compounds were noted for higher binding free energy, except Cynaroside, in the selective pocket of JEV–RdRp against reference compounds as a function of 100 ns interval.

## 4. Conclusions

Japanese encephalitis virus–RNA-dependent RNA polymerase (JEV–RdRp) is a potential target for antiviral drugs, as they are responsible for viral genome replication. The purpose of this study was to identify potent antiviral compounds from *E. angustifolia* by inhibiting JEV–RdRp using in silico approach. Based on the substantial docking energy (>−10 kcal/Mol) and pharmacokinetics analysis, six compounds—Echinacoside, Echinacin, Rutin, Cynaroside, Quercetagetin 7-glucoside, and Kaempferol-3-glucoside—were selected. Based on the hydrogen, hydrophobic, and other molecular interaction analyses, the stability of the respective compounds with JEV–RdRp’s binding site was studied. The final confirmation of the stability of the protein–ligand complex was made with the analysis of the MD simulation and post-simulation analysis. In conclusion, the results revealed that the selected compounds from *E. angustifolia* are acceptable inhibitors of the JEV–RdRp protein and can be utilized for further studies for developing a potential antiviral drug against Japanese encephalitis.

## Figures and Tables

**Table 1:** Redocking scores and intermolecular interaction analysis for the potent natural compounds against JEV–RdRp extracted at 4 Å around the docked ligand.

S. No.	Compound	Docking Score (kcal/mol)	H-Bond	π–π/π–Cation Stacking	Hydrophobic	Polar	Negative	Positive	Glycine	Salt Bridge
a.	Echinacoside	−11.1	Arg^460^(2), Glu^461^, Ser^604^(2), Ser^666^, Asp^669^	Arg^474^	Ile^476^, Val^509^, Tyr^610^, Phe^713^, Cys^714^, Trp^800^, Ile^802^	Ser^473^, Ser^604^, Thr^609^, Asn613, Ser^666^, Ser^801^, His^803^	Glu^461^, Asp^668^, Asp^669^	Arg^460^, Lys^462^, Lys^463^, Lys^471^, Arg^474^, Arg^742^	Gly^412^, Gly^472^, Gly^605^, Gly^667^	-
b.	Echinacin	−10.4	Leu^411^, Tyr^610^, Cys^714^, Ser^715^, Arg^734^, Arg^742^, Ser^801^	-	Leu^411^, Ala^413^, Ile^476^, Val^607^, Tyr^610^, Cys^714^, Trp^800^, Ile^802^	Ser^604^, Gln^606^, Thr^609^, Ser^715^, Asn^716^, Ser^799^ Ser^801^, His^803^	Glu^738^	Arg^460^, Arg^474^, Arg^734^, Arg^742^,	Gly^412^	-
c.	Rutin	−10.4	Lyn^463^, Ala^475^, Ser^604^, Gln^606^, Ser^666^, Asp^668^	Tyr^610^	Ala^413^, Val^414^, Ala^475^, Ile^476^, Trp^477^, Val^607^, Tyr^610^, Cys^714^, Trp^800^, Ile^802^	Ser^604^, Gln^606^, Thr^609^, Ser^666^, Ser^715^, Ser^801^, His^803^	Asp^668^, Asp^669^	Arg^460^, Lys^471^, Arg^474^, Arg^742^	Gly^412^, Gly^605^, Gly^667^	-
d.	Cynaroside	−10.0	Ser^666^, Asp^668^, Asp^669^, His^803^	-	Leu^411^, Ala^413^, Val^607^, Tyr^610^, Ala^611^, Cys^714^, Ile^802^	Asn^495^, Ser^604^, Gln^606^, Thr^609^, Asn^613^, Ser^666^, Ser^801^, His^803^	Glu^510^, Asp^668^, Asp^669^	Lys^404^, Lys^471^, Hip^498^	Gly^412^, Gly^605^, Gly^667^	-
e.	Quercetagetin 7-glucoside	−10.0	Ser^666^, Asp^668^, Ser^801^	-	Leu^411^, Ala^413^, Val^607^, Tyr^610^, Ala^611^, Cys^714^, Ile^802^	Asn^495^, Ser^604^, Gln^606^, Thr^609^, Asn^613^, Ser^666^, Ser^801^, His^803^	Glu^510^, Asp^668^, Asp^669^	Lys^404^, Lys^471^, Arg^474^	Gly^412^, Gly^605^, Gly^667^	-
f.	Kaempferol-3-glucoside	−10.0	Asp^541^, Ser^604^, Ser^801^	-	Leu^411^, Ala^413^, Trp^540^, Val^607^, Tyr^610^, Trp^800^, Ile^802^	Asn^495^, Ser^604^, Gln^605^, Thr^609^, Asn^613^, Ser^666^, Ser^801^	Asp^541^, Asp^668^	Arg^474^	Gly^412^, Gly^667^,	-
G	Adenosine triphosphate	−8.6	Ser^604^, Gln^606^, Ser^715^, Arg^742^, Trp^800^, Ile^802^	--	Leu^411^, Ala^413^, Val^607^, Tyr^610^, Trp^800^, Ile^802^	Ser^604^, Gln^606^, Thr^609^, Ser^715^, Ser^799^, Ser^801^	-	Arg^460^, Lys^463^, Lys^471^, Arg^474^, Arg^734^, Arg^742^	Gly^412^, Gly^605^	Lys^463^, Arg^474^, Arg^734^, Arg^742^
h	Guanosine-5′-triphosphate	−9.0	Asp^541^, Asn^613^, Asp^668^, Ser^801^, Ile^802^	Tyr^610^	Trp^540^, Tyr^610^, Trp^800^, Ile^802^	Ser^604^, Asn^613^, Thr^609^, Ser^666^, Ser^801^, His^803^	Asp^541^, Asp^668^, Asp^669^	Arg^460^, Lys^463^, Arg^474^, Arg^742^	Gly^667^	Arg^460^, Lys^463^, Arg^474^, Arg^742^

**Table 2:** Calculated net binding free energy for the selected docked poses of JEV–RdRp natural compounds snapshots from the last 10 ns interval of 100 ns MD simulation.

Energy (kcal/mol)	JEV–RdRP Poses with Natural Compounds from *Echinacea angustifolia*							
	Echinacoside	Echinacin	Rutin	Cynaroside	Quercetagetin 7-Glucoside	Kaempferol-3-Glucoside	Adenosine Triphosphate	Guanosine-5′-Triphosphate
Δ*G*_Bind_	−80.15 ± 8.04	−81.67 ± 8.31	−80.33 ± 5.54	−57.26 ± 4.13	−66.24 ± 5.6	−62.13 ± 5.97	−69.62 ± 8.62	−47.98 ± 11.41
Δ*G*_BindCoulomb_	−57.04 ± 8.1	−32.93 ± 8.82	−41.87 ± 5.69	−28.94 ± 6.36	−30.43 ± 6.17	−28.68 ± 5.86	−242.97 ± 54.21	−242.62 ± 52.45
Δ*G*_BindCovalent_	4.62 ± 2.38	4.17 ± 2.11	5.19 ± 2.51	6.06 ± 2.1	4.29 ± 1.87	3.94 ± 1.67	7.83 ± 4.45	5.13 ± 2.45
Δ*G*_BindHbond_	−7.11 ± 1	−3.95 ± 0.55	−6.11 ± 0.69	−3.68 ± 0.79	−4.56 ± 0.92	−3.46 ± 0.63	−15.77 ± 2.08	−10.65 ± 2.24
Δ*G*_BindLipo_	−18.26 ± 2.1	−16.1 ± 1.22	−17.43 ± 1.37	−13.59 ± 1.49	−16.4 ± 1.04	−16.22 ± 1.34	−5.09 ± 0.58	−3.56 ± 0.61
Δ*G*_BindPacking_	−2.04 ± 0.63	−7.16 ± 1.27	−0.71 ± 0.17	−3.35 ± 0.62	−2.07 ± 0.35	−2.38 ± 0.76	−0.15 ± 0.28	−1.48 ± 0.61
Δ*G*_BindSolvGB_	66.69 ± 6.11	41.52 ± 3.6	46.21 ± 3.44	42.08 ± 3.18	42.84 ± 4.72	39.37 ± 3.82	224.6 ± 49.37	236.56 ± 49.6
Δ*G*_BindvdW_	−67 ± 4.05	−67.22 ± 3.38	−65.54 ± 3.71	−55.81 ± 3.28	−59.9 ± 3.17	−54.7 ± 3.41	−38.07 ± 4.69	−31.35 ± 4.63
Ligand Strain Energy	11.92 ± 3.73	7.91 ± 2.37	10.64 ± 3.46	6.0 ±2.13	5.2 ± 2.19	5.57 ± 2.51	7.55 ± 4.06	3.99 ± 2.95

## Data Availability

The datasets used and/or analyzed during the current study are available from the corresponding author on reasonable request.

## Supplementary Materials

The following supporting information can be downloaded at: https://www.mdpi.com/article/10.3390/life12070952/s1.
