# Supplementary material for: Discovery of Small Molecules from Echinacea angustifolia Targeting RNA-Dependent RNA Polymerase of Japanese Encephalitis Virus
Source: Life (Basel). 2022 Jun 24;12(7):952. doi: 10.3390/life12070952 (PMC9324244; doi:10.3390/life12070952)
Supplement: Supplementary file 1 [file life-12-00952-s001.zip › life-1756996-supplementary.pdf]

Pardeep Yadav[1,5], Sherif A. El-Kafrawy[2,3], Mai M. El-Day[2,3], Wejdan T. Alghafari [2,4], Arwa A. Faizo[2,3], Saurabh Kumar Jha[1], Vivek Dhar Dwivedi[5,6*], Esam I. Azhar[2,3*]

[1]Department of Biotechnology, School of Engineering & Technology, Sharda University, Greater Noida, Uttar Pradesh, 201310, India

[2]Special Infectious Agents Unit-BSL-3, King Fahd Medical Research Center, King Abdulaziz University, Jeddah-21362, Saudi Arabia

[3]Department of Medical Laboratory Sciences, Faculty of Applied Medical Sciences, King Abdulaziz University, Jeddah-21362, Saudi Arabia

[4]Clinical Nutrition Department, Faculty of Applied Medical Sciences, King Abdulaziz University, Jeddah-21362, Saudi Arabia

[5]Center for Bioinformatics, Computational and Systems Biology, Pathfinder Research and Training Foundation, Greater Noida-201308, India

[6]Institute of Advanced Materials, IAAM, 59053 Ulrika, Sweden

Corresponding Authors:
V.D.D., Email: vivek.dwivedi@iaam.se

E.I.A., Email: eazhar@kau.edu.sa

S1. Results

S1.1. Structure-based virtual screening

Table S1. List of compounds from *Echinacea angustifolia* and binding affinities calculated against JEV^{RdRp} domain using PyRx 0.8 virtual screening tool.

S.no.	Pubchem CID	Compound names	Binding affinity (Kcal/mol)
1	5281771	Echinacoside	-11.1
2	6439941	Echinacin	-10.5
3	5280805	Rutin	-10.4
4	5280637	Cynaroside	-10
5	44259796	Quercetagetin 7-glucoside	-10
6	5282102	Kaempferol-3-glucoside	-9.4
7	6537500	Cynarin	-9.3
8	5280794	Stigmasterol	-9.3
9	5991	Ethynylestradiol	-9
10	11101	2-nitrobenzaldehyde	-9
11	5315832	Isochlorogenic acid	-8.8
12	5281769	Cynarin	-8.8
13	5134221	Cichoric acid	-8.8
14	222284	Beta-sitosterol	-8.7
15	5280445	Luteolin	-8.6
16	1794427	Chlorogenic acid	-8.6
17	5280343	Quercetin	-8.5
18	5281654	Isorhamnetin	-8.4
19	5280863	Kaempferol	-8.4
20	5280443	Apigenin	-8.4
21	6440397	Caftaric acid	-7.9
22	440946	Levan N	-7.8
23	11587035	Echinamine b	-7.7
24	131751109	Neoherculin	-7.1
25	5281515	Beta-caryophyllene	-6.9
26	14350	Caryophyllene epoxide	-6.9
27	5988	Sucrose	-6.8
28	91723653	Isogermacrene d	-6.7
29	5280934	Linolenic acid	-6.5
30	6443006	Dodeca-2e,4e-dienoic acid isobutylamide	-6.3
31	593849	N-(2-methylpropyl)undeca-2,4-dien-8,10-diynamide	-6.3
32	3931	Octadeca-9,12-dienoic acid	-6.3
33	441552	1-tridecene-3,5,7,9,11-pentayne	-6.1
34	93009	Bornyl acetate	-6
35	528755	Tridec-1,3-diene-5,7,9,11-tetrayne	-6
36	16219508	Beta-d-arabino-hex-2-ulofuranosyl d-gluco-hexopyranoside	-6
37	11413953	Dodeca-2E,4E,8Z,10Z-Tetraenoic Acid Isobutylamide	-6
38	6441887	Echinolone	-5.8
39	149801	Isotussilagine	-5.8
40	64685	Borneol	-5.6
41	5322026	(11E)-trideca-1,11-dien-3,5,7,9-tetrayne	-5.6
42	22041880	[(2E,4E)-dodeca-2,4-dienyl] 3-methylbutanoate	-5.6
43	12811248	8z-pentadecen-2-one	-5.5
44	11005	Tetradecanoic acid	-5.5
45	10469	Hexacosanoic acid	-5.4
46	68972	triacontan-1-ol	-5.3
47	985	Palmitic acid	-5.2
48	25913	1-pentadecene	-5.2
49	445639	Oleic acid	-5
50	22199	Ethyl docosanoate	-5
51	247	Betaine	-4.5
52	81653	Dimethyl-isobutyl amine	-3.5
53	6354	Oxirane	-2.3

S1.2. ADME profiling

Table S2. ADME profiling of the selected natural compounds against binding pocket of JEV^{RdRp}.

Properties	Echinacoside	Echinacin	Rutin	Cynaroside	Quercetagetin 7-glucoside	Kaempferol-3-glucoside	Adenosine triphosphate	Guanosine-5'-triphosphate
LogS	-1.243	-4.214	-3.928	-3.812	-3.498	-3.878	-0.543	-0.625
LogD	0.447	2.67	0.695	0.844	-0.058	1.124	-1.539	-2.101
LogP	-1.514	3.481	-0.763	0.317	-0.456	-0.02	-3.918	-3.931
Pgp-inh	0.001	0.002	0.002	0.001	0.007	0.003	0	0
Pgp-sub	0.962	0.835	0.978	0.895	0.7	0.514	0.556	0.011
HIA	1	0.761	0.925	0.864	0.872	0.627	0.998	0.782
F(20%)	0.975	0.882	0.234	0.842	0.484	0.23	0.999	0.999
F(30%)	1	0.999	0.999	0.999	0.999	0.995	1	1
Caco-2	-6.559	-6.106	-6.336	-6.126	-6.359	-6.105	-6.497	-6.627
MDCK	0.000209	1.02E-05	2.97E-05	2.94E-05	1.01E-05	1.44E-05	2.91E-05	0.000107
BBB	0.332	0.012	0.111	0.061	0.012	0.074	0.76	0.693
PPB	86.35%	100.46%	83.81%	87.42%	87.84%	87.82%	14.13%	39.03%
VDss	0.195	0.61	0.754	0.879	0.864	0.946	0.503	0.412
Fu	29.06%	1.66%	20.87%	11.59%	16.08%	14.66%	74.65%	70.39%
CYP1A2-inh	0.001	0.16	0.013	0.087	0.038	0.07	0	0
CYP1A2-sub	0.007	0.032	0.026	0.048	0.026	0.037	0.214	0.307
CYP2C19-inh	0.012	0.245	0.011	0.017	0.007	0.016	0.049	0.031
CYP2C19-sub	0.049	0.053	0.05	0.054	0.045	0.053	0.025	0.021
CYP2C9-inh	0	0.597	0.002	0.012	0.007	0.012	0.002	0.005
CYP2C9-sub	0.355	0.938	0.246	0.205	0.084	0.621	0.591	0.078
CYP2D6-inh	0.001	0.649	0.007	0.049	0.006	0.082	0.022	0
CYP2D6-sub	0.151	0.535	0.155	0.209	0.139	0.187	0.04	0.022
CYP3A4-inh	0.009	0.44	0.013	0.059	0.018	0.062	0.003	0.004
CYP3A4-sub	0.003	0.091	0.003	0.019	0.005	0.018	0.001	0.01
CL	0.301	4.242	1.349	4.318	3.231	3.554	1.346	1.712
T12	0.605	0.541	0.524	0.631	0.901	0.725	0.9	0.925
hERG	0.014	0.136	0.017	0.027	0.085	0.024	0.006	0.003
H-HT	0.117	0.073	0.092	0.081	0.122	0.113	0.255	0.901
DILI	0.065	0.844	0.982	0.95	0.986	0.979	0.935	0.992
Ames	0.185	0.686	0.805	0.757	0.813	0.775	0.078	0.989
ROA	0.082	0.033	0.05	0.034	0.024	0.111	0.042	0.107
FDAMDD	0.003	0.122	0.014	0.031	0.008	0.011	0.937	0.654
SkinSen	0.146	0.856	0.036	0.346	0.51	0.057	0.328	0.964
Carcinogenicity	0.056	0.685	0.064	0.569	0.053	0.095	0.466	0.679
EC	0.003	0.003	0.003	0.003	0.003	0.003	0.003	0.003
EI	0.004	0.022	0.01	0.031	0.081	0.015	0.012	0.045
Respiratory	0.006	0.027	0.015	0.061	0.017	0.024	0.837	0.906
BCF	0.868	0.916	0.938	0.819	0.929	0.771	0.269	-0.117
IGC50	4.198	5.115	3.968	4.149	3.861	3.905	2.789	3.985
LC50	5.001	5.818	4.92	4.845	4.441	4.732	4.129	4.586
LC50DM	5.535	6.081	5.517	5.437	5.436	5.136	4.156	5.14
NR-AR	0.003	0.016	0.264	0.012	0.022	0.16	0.006	0.003
NR-AR-LBD	0.611	0.866	0.636	0.277	0.329	0.536	0.001	0.002
NR-AhR	0.156	0.916	0.835	0.882	0.884	0.906	0	0
NR-Aromatase	0.879	0.923	0.914	0.914	0.901	0.938	0	0
NR-ER	0.216	0.878	0.209	0.273	0.262	0.374	0.027	0.029
NR-ER-LBD	0.098	0.938	0.127	0.17	0.206	0.566	0.007	0.004
NR-PPAR-gamma	0.954	0.979	0.953	0.932	0.942	0.905	0.002	0.002
SR-ARE	0.211	0.937	0.125	0.476	0.485	0.32	0.001	0.001
SR-ATAD5	0.89	0.737	0.586	0.428	0.374	0.234	0.014	0.004
SR-HSE	0.862	0.896	0.405	0.639	0.405	0.191	0.001	0
SR-MMP	0.862	0.971	0.763	0.745	0.825	0.767	0.003	0.005
SR-p53	0.688	0.969	0.74	0.835	0.861	0.853	0.007	0.004
MW	786.26	578.14	610.15	448.1	480.09	448.1	507	519.97
Vol	725.847	555.862	552.318	413.147	430.728	413.147	374.04	384.795
Dense	1.083	1.04	1.105	1.085	1.115	1.085	1.355	1.351
nHA	20	12	16	11	13	11	18	19
nHD	12	6	10	7	9	7	5	4
TPSA	324.44	196.35	269.43	190.28	230.74	190.28	270.38	290.02
nRot	14	8	6	4	4	4	8	12
nRing	5	5	5	4	4	4	3	2
MaxRing	6	10	10	10	10	10	9	9
nHet	20	12	16	11	13	11	21	22
fChar	0	0	0	0	0	0	0	-1
nRig	32	32	30	24	24	24	22	19
Flex	0.438	0.25	0.2	0.167	0.167	0.167	0.364	0.632
nStereo	15	5	10	5	5	5	4	3
Toxicophores	4	2	2	2	2	1	1	2
Acute_Aquatic_Toxicity	5	5	2	2	2	2	0	0
LD50_oral	0	0	0	0	0	0	0	1
NonGenotoxic_Carcinogenicity	1	1	0	0	0	0	0	0
Skin_Sensitization	9	5	8	7	10	4	0	2
SureChEMBL	0	0	0	0	0	0	1	2
Genotoxic_Carcinogenicity_Mutagenicity	1	1	0	0	0	0	0	0
NonBiodegradable	2	1	2	2	2	2	1	1
QED	0.054	0.138	0.14	0.261	0.208	0.279	0.26	0.172
Synth	5.372	4.081	4.783	3.924	4.143	3.884	6.145	6.162
Fsp3	0.571	0.2	0.444	0.286	0.286	0.286	0.5	0.3
MCE-18	131.182	101.611	122.949	87.63	94.37	87.63	91.867	44
Natural Product-likeness	1.605	1.535	2.015	1.972	2.164	2.073	0.651	0.079
Alarm_NMR	3	3	3	3	3	2	0	1
BMS	1	0	0	0	1	0	0	2
Chelating	1	0	1	1	3	0	0	0
PAINS	1	0	1	1	1	0	0	0
Lipinski	Rejected	Rejected	Rejected	Rejected	Rejected	Rejected	Rejected	Rejected
Pfizer	Accepted	Accepted	Accepted	Accepted	Accepted	Accepted	Accepted	Accepted
GSK	Rejected	Rejected	Rejected	Rejected	Rejected	Rejected	Rejected	Rejected
GoldenTriangle	Rejected	Rejected	Rejected	Accepted	Accepted	Accepted	Rejected	Rejected

S1.3. Redocking and molecular contact analysis

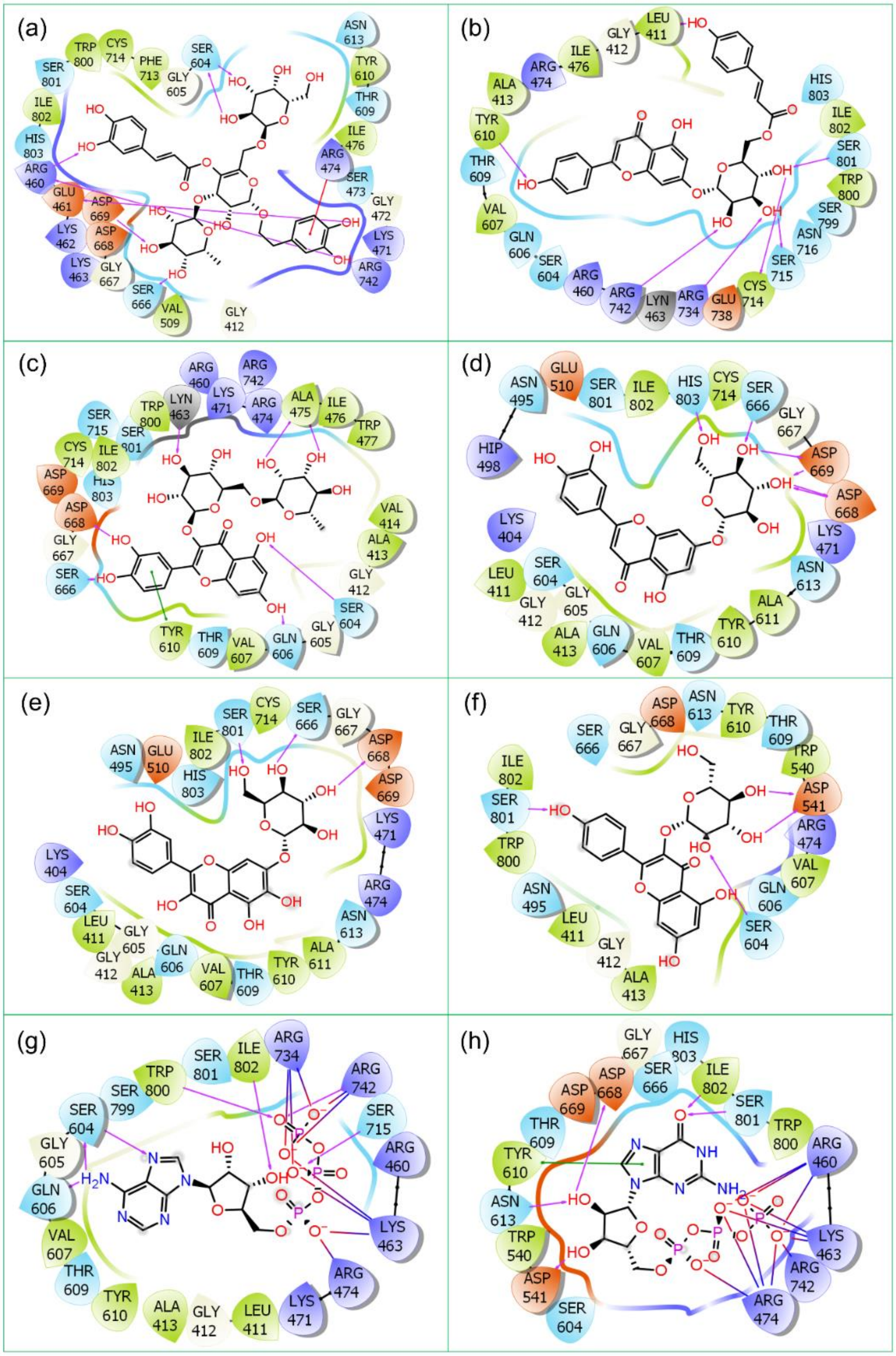

Figure S1. Two-dimensional (2D) poses of the selected natural compounds: (**a**) Echinacoside, (**b**) Echinacin, (**c**) Rutin, (**d**) Cynaroside, (**e**) Quercetagetin 7-glucoside, and (**f**) Kaempferol-3-glucoside, with comparison to the reference compounds (**g**) ATP and (**h**) GTP in the selected binding pocket of JEV^{RdRp} showing intermolecular interactions with residues extracted at 4 Å around the docked ligand. In 2D interaction maps, pink arrow (H-bond), green line (π–π stacking, red-violet (salt bridge), red (negative), violet (positive), green (hydrophobic), and blue (polar) color residues exhibit the interactions in the respective docked complexes.

S1.4. Molecular Dynamics simulation analysis

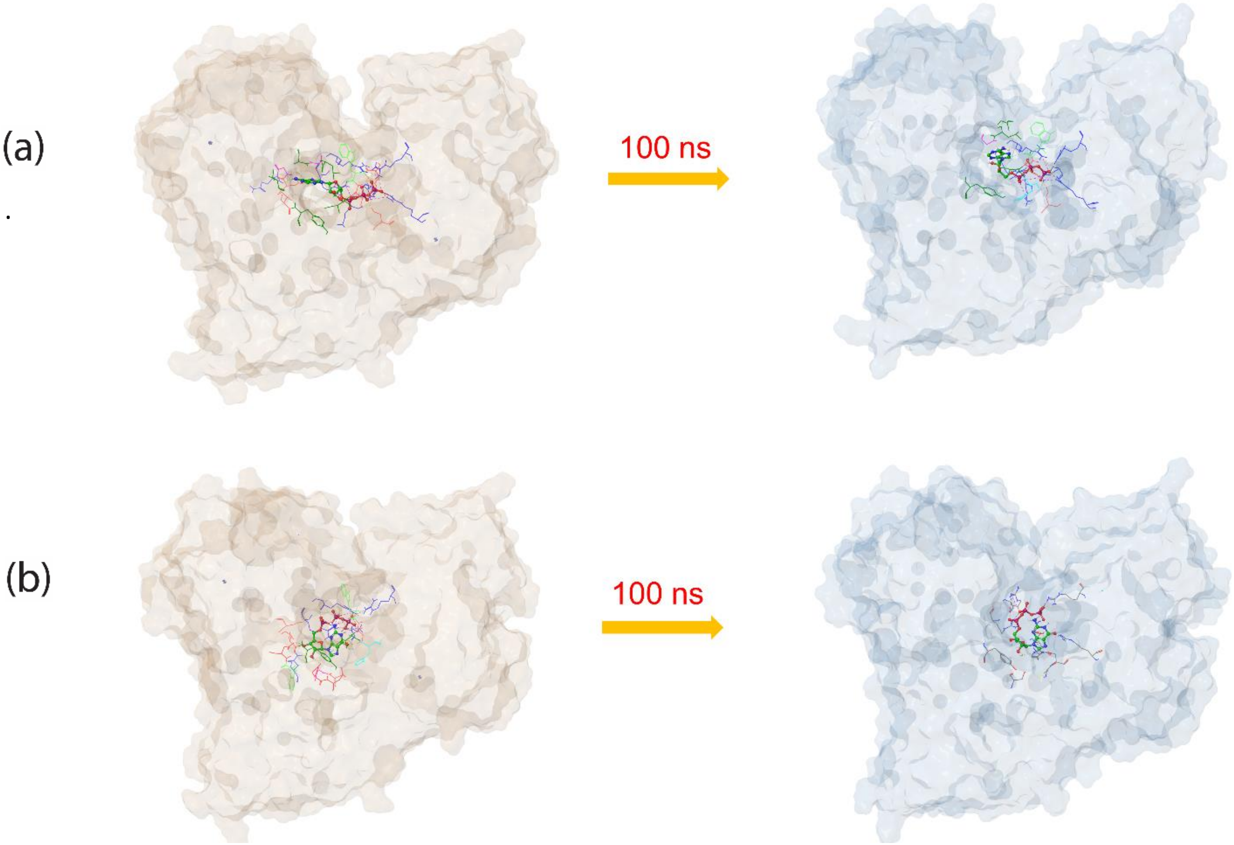

Figure S2. Representation of docked Reference compounds (**a**) ATP and (**b**) GTP in the selective binding pocket of JEV–RdRp before and after the 100 ns MD simulation.

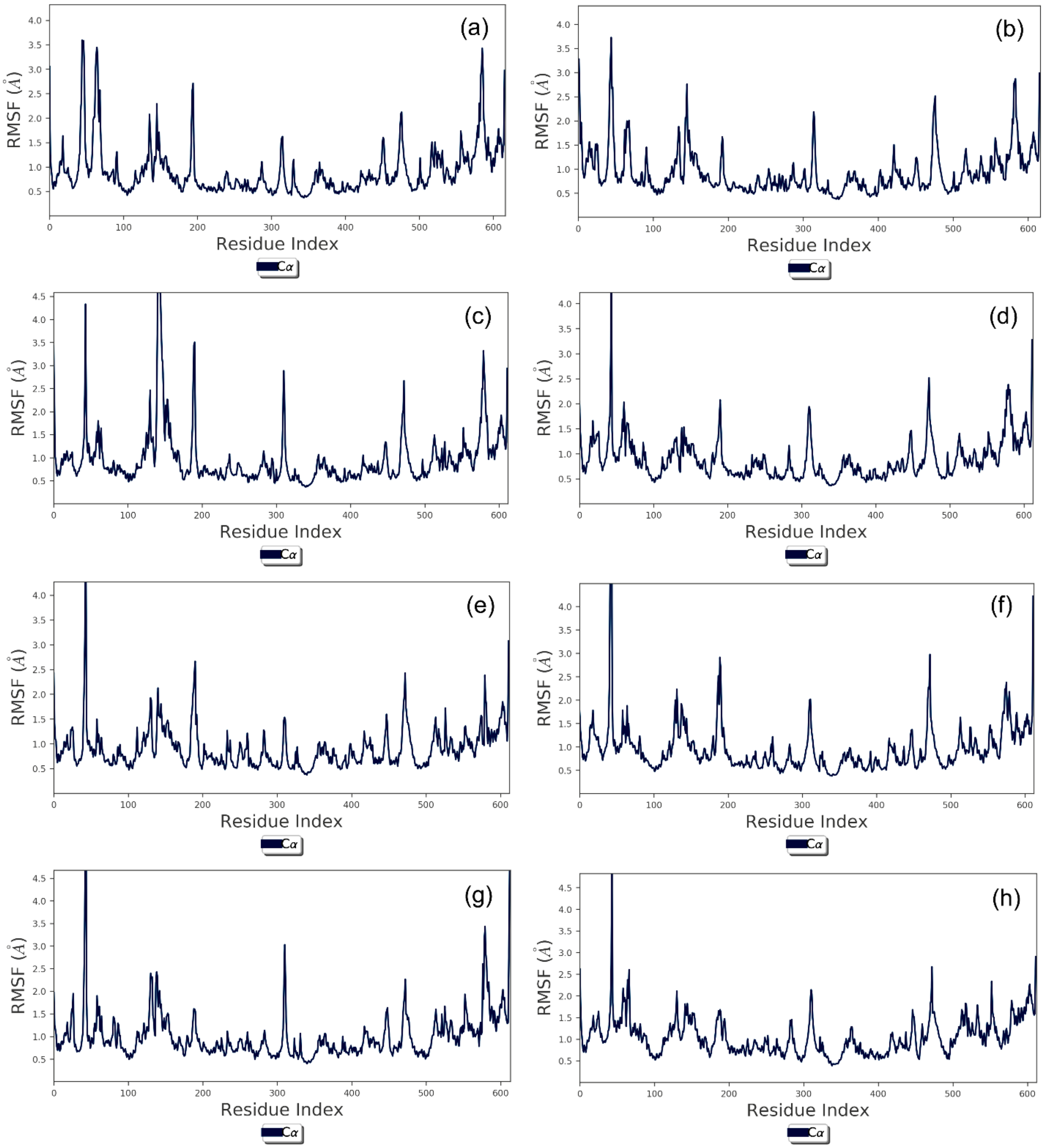

Figure S3. RMSF plot for the JEV^{RdRP} docked with selected natural compounds: (**a**) Echinacoside, (**b**) Echinacin, (**c**) Rutin, (**d**) Cynaroside, (**e**) Quercetagetin 7-glucoside, and (**f**) Kaempferol-3-glucoside, with comparison to the reference compounds (**g**) ATP and (**h**) GTP.

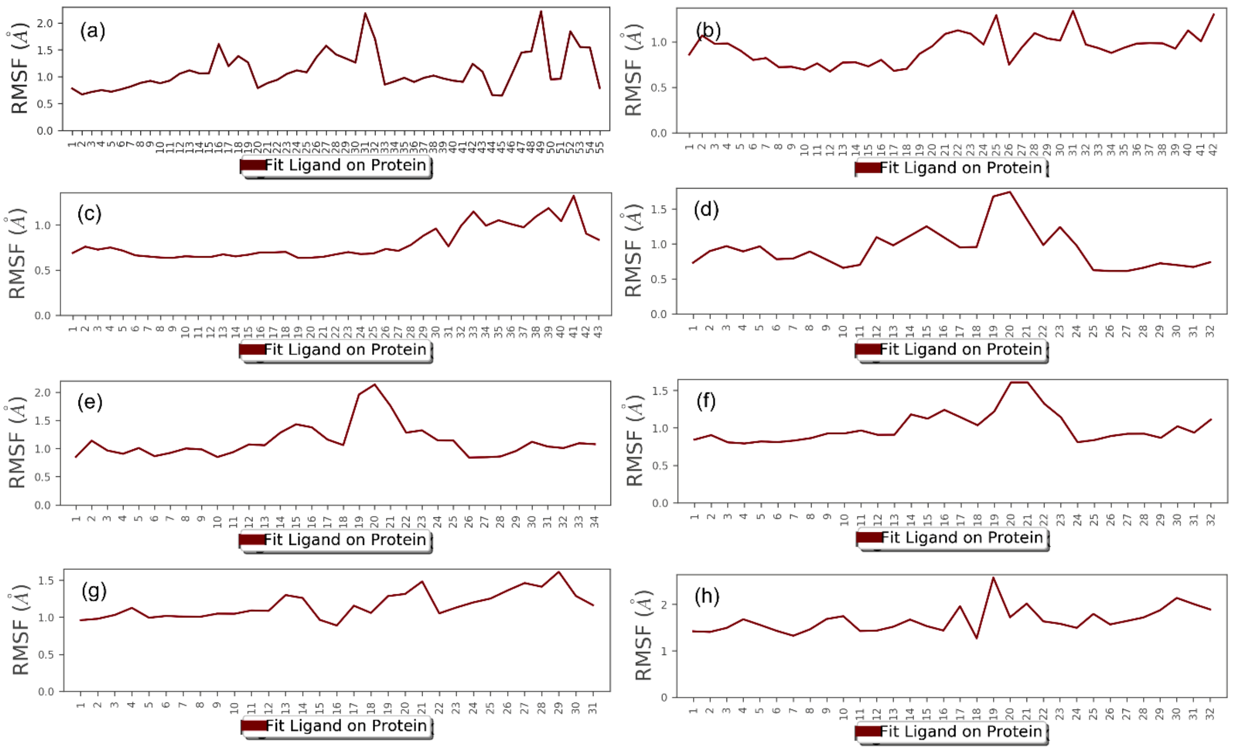

Figure S4. RMSF plot for the selected natural compounds: (**a**) Echinacoside, (**b**) Echinacin, (**c**) Rutin, (**d**) Cynaroside, (**e**) Quercetagetin 7-glucoside, and (**f**) Kaempferol-3-glucoside, with comparison to the reference compounds (**g**) ATP and (**h**) GTP.

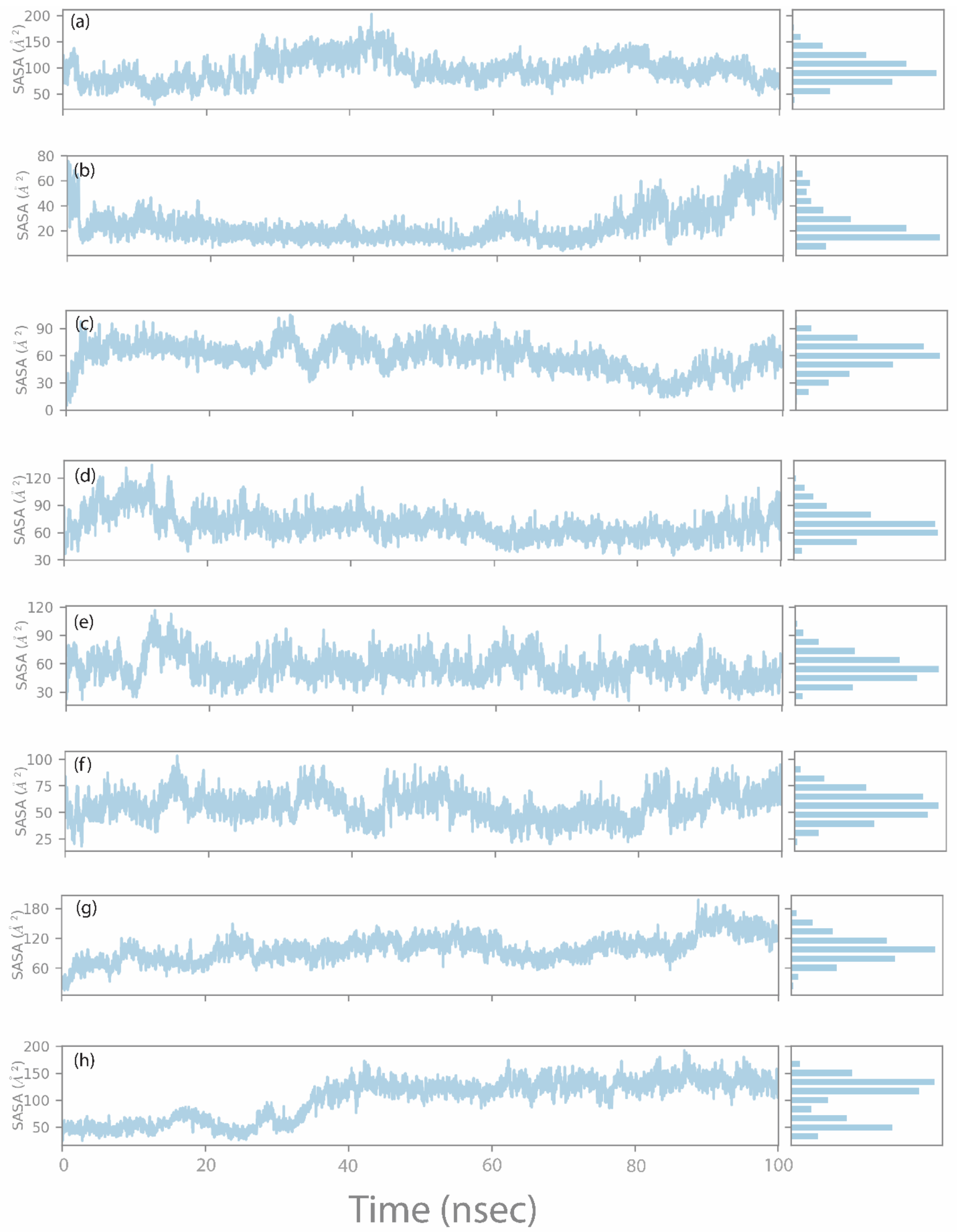

Figure S5. Solvent accessible surface area (SASA) of the selected natural compounds: (**a**) Echinacoside, (**b**) Echinacin, (**c**) Rutin, (**d**) Cynaroside, (**e**) Quercetagetin 7-glucoside, and (**f**) Kaempferol-3-glucoside, with comparison to the reference compounds (**g**) ATP and (**h**) GTP.

S1.6. Post-simulation analysis

S1.6.1. Principal component analysis

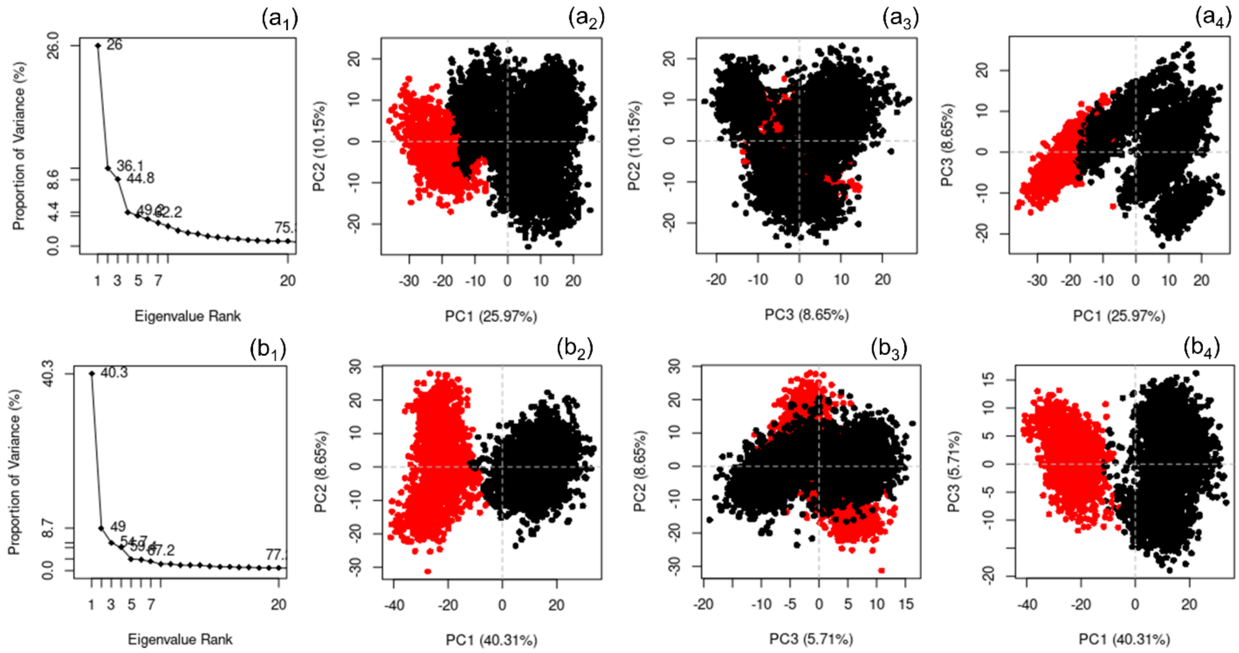

Figure S6. Principal component analysis for the generated molecular dynamics trajectories of JEV–RdRp docked with reference compounds (**a**) ATP and (**b**) GTP. The percentage of total mean square displacement of residual positional variations recorded in each dimension is categorized by equivalent eigenvalues or PCs.
